# Hyperoside Ameliorates Diabetic Retinopathy *via* Anti-Oxidation, Inhibiting Cell Damage and Apoptosis Induced by High Glucose

**DOI:** 10.3389/fphar.2020.00797

**Published:** 2020-05-29

**Authors:** Wei Wu, Zhaolu Xie, Qing Zhang, Yunqi Ma, Xiaoting Bi, Xue Yang, Bin Li, Jianhong Chen

**Affiliations:** Department of Pharmacy, Daping Hospital, Army Medical University, Chongqing, China

**Keywords:** hyperoside, apoptosis, proliferation, oxidative stress, diabetic retinopathy, retinal vascular endothelium

## Abstract

**Background:**

Hyperoside (Hyp) is a flavonoid substance extracted from plants, which has the functions of anti-cancer, anti-inflammatory, and anti-oxidation. In the previous study, we found that Hyp reduced the injury of rat retinal vascular endothelial cells (RVECs) induced by H_2_O_2_.

**Method:**

In the present research, we evaluated the protective effect of Hyp on the pathological damage of retina caused by high glucose of diabetes mellitus (DM) in *in vitro* and *in vivo* experiments. The effect of Hyp on cell viability, oxidative stress level, and apoptosis of RVECs was assessed.

**Results:**

Hyp significantly reduced the of RVECs damage, oxidative stress level, and cell apoptosis induced by high glucose *in vitro*. In DM model rats, Hyp treatment could significantly reduce blood glucose levels and the pathological damage of retina caused by DM and increase the proliferation of RVECs while exerting the inhibition on apoptotic activity. Furthermore, Hyp treatment decreased the expressions of apoptotic proteins including caspase-3, caspase-9, and Bax in RVECs of DM rats, while increased the expression of anti-apoptotic protein Bcl-2.

**Conclusion:**

Hyp may have protective effect on diabetes-induced retinopathy by reducing oxidative stress, inhibiting cell damage, and apoptosis induced by high glucose.

## Introduction

Diabetes is a metabolic disease which can lead to systematic complications including retinopathy, cardiovascular complications, and diabetic nephropathy. Among these complications, diabetic retinopathy can eventually lead to irreversible loss of vision or blindness. Currently, anti-vascular endothelial growth factor (VEGF) reagents and corticosteroids are broadly used in the clinic for treating this disease (Singer et al., 2016). Calcium dobesilate, which is an established vasoactive and angioprotective drug, has been used to treat diabetic retinopathy at the systematic and local ocular level with promising results (Haritoglou et al., 2009; Cai et al., 2017). However, these drugs may show some side effects, and not all patients respond to anti-VEGF agents. Therefore, it is necessary to further explore and develop new medicines to treat diabetic retinopathy.

Many studies show that medicinal plants are important resources for new drug research and development (Peng et al., 2018). Some studies have demonstrated that flavonoids have beneficial effects in treating diabetic complications (Testa et al., 2016). Based on many experiments, flavonoids have obvious anti-inflammation and anti-oxidative stress activities, which are highly beneficial in treating diabetic retinopathy. For example, polyphenol from cocoa could enhance the retinal silent mating type information regulation 2 homolog 1 (SIRT1) pathway in streptozotocin (STZ) induced-diabetic rats, thereby protecting the retina from oxidative stress damage in diabetes mellitus (Duarte et al., 2015). Treatment of another anti-oxidative flavonoid, eriodictyol, significantly attenuated inflammation and plasma lipid peroxidation in the retina, and preserved the integrity of blood-retinal barrier (BRB) (Bucolo et al., 2012).

Hyperoside (Hyp) is a flavonoid extracted from Hypericum and Crataegus species. The anti-oxidative activity of Hyp has been confirmed (Luna-Vazquez et al., 2013; El Hosry et al., 2016; Hao et al., 2016). Further studies have shown that Hyp also has anti-inflammatory (Sun et al., 2002) and anti-cancer (Ogawa et al., 2001; Hao et al., 2016) effects. In the STZ induced-diabetic rats, treatment of Hyp at 25 and 50 mg/kg doses for 30 days exhibited significant antihyperglycemic effect (Verma et al., 2013). This study also showed that Hyp could improve the function of pancreatic islets, increase glycolysis, and decrease gluconeogenesis. Regarding to diabetes-related complications, the protective effects of Hyp had been shown on diabetic nephropathy at early stage in a STZ induced-diabetic mouse model (Cai et al., 2017). Our previous results showed that Hyp could reduce the damage of isolated rat retinal vascular endothelial cells (RVECs) induced by H_2_O_2_ (Wu et al., 2013). However, the effect of Hyp on diabetic retinopathy and its possible mechanism remains to be elucidated. Therefore, this study was undertaken to examine the protective effect of Hyp on the damage of retina caused by high glucose of diabetes mellitus in *in vitro* and *in vivo* experiments, and its probable molecular mechanism.

## Materials and Methods

### Chemicals and Reagents

Hyperoside (purity≥98%) was purchased from Nanjing Zelang Medical Technological Co., Ltd (Nanjing, China). Superoxide Dismutase (SOD) and malondialdehyde (MDA) detection kits were purchased from Nanjing Jiancheng Bioengineering Institute (Nanjing, China). Reactive Oxygen Species (ROS) Assay Kit was purchased from Beyotime Institute of Biotechnology (Jiangsu, China). In situ cell death detection kit (12156792910) was purchased from Roche Diagnostics (Mannheim, Germany). All primers were synthesized by Invitrogen Corporation (Chengdu, China). SYBR Green real-time PCR Master Mix kit was purchased from Toyobo Corporation (Osaka, Japan). Anti-von Willebrand factor (vWF) antibody (ab201336) was purchased from Abcam (Cambridge, UK). Anti-Bax (sc-4239), anti-Bcl-2 (sc-509), anti-Cyto C (sc-13561), anti–caspase-3 (sc-7148), anti–caspase-9 (sc-81663) antibody was purchased from Santa Cruz Biotechnology (Santa Cruz, CA, USA). HRP-conjugated goat anti-rabbit IgG (SA00001-2) or goat anti-mouse IgG (SA00001-1) was purchased from ProteinTech (Chicago, IL, USA).

### Isolation, Culture, and Identification of Rat Retinal Vascular Endothelial Cells

Primary rat retinal vascular endothelial cells (RVECs) was obtained from retinas as described previously (Matsubara et al., 2000; Suzuki et al., 2003). Briefly, rats were sacrificed, eyes were removed rapidly, and placed into sterile HBSS solution supplemented with 1,000 U tobramycin for 30 min. Eyes tissues were separated 0.5 mm from the back of limbus corneae by cutting. After enucleation, tissues were rinsed with HBSS to eliminate the remaining retinal pigment epithelium. The retinas were finely cut into small pieces in tissue culture dishes and digested with three volumes of 0.25% collagenase for 90 min at room temperature. The mixture was filtered through 100-μm nylon mesh and centrifuged at 300*g* for 5 min. The pellets were re-suspended into DMEM supplemented with 10% FBS and seeded into fibronectin-coated dishes, and subsequently kept in 37°C incubator with 5% CO_2_. The cultured cells were identified by immunofluorescence staining using anti-vWF antibody.

### Cell Viability, SOD Activity, MDA, and ROS Level of RVECs Induced by High Glucose

RVECs were adherent cultured in 96-well plates. After 6 h culture, the cells were pretreated with high glucose (30 mmol/L) for 24 h, then were incubated with Hyp (10 μg/ml) for 72 h. At 24, 48, and 72 h, respectively, 20 μl thiazolyl tetrazolium (MTT) solution (5 mg/ml, Roche) was added into each well in a total volume of 200 μl, and incubated for 4 h under dark. Thereafter, the supernatant was removed, DMSO (150 μl/well) was added to dissolve the produced formazan crystals. The absorbance was measured at 490 nm by multi-label Counter (Biotek Synergy 2). To measure the cell viability as a percentage, the absorbance of each sample was divided into the absorbance of the control and multiplied by 100. In the assay of SOD and MDA, the supernatant of cell culture were collected at 72 h after Hyp treatment. SOD activity and MDA level were measured according to the kit operation instruction. The ROS production was detected using 2′,7′-dichlorofluorescein diacetate (DCFH-DA) by flow cytometry. After the treatments, RVECs were harvested and resuspended in a serum-free medium containing DCFH-DA (10μM). After incubating for 30min at 37°C and washing with the serum-free medium, the fluorescent intensity of RVECs was measured using flow cytometry at 488nm excitation wavelength and 525nm emission wavelength.

### Bax, Bcl-2, CytC, Caspase-9, and Caspase-3 mRNA Expression of RVECs Induced by High Glucose

As the group and treatment mentioned above, cultured cells were preincubated with high glucose (30 mmol/L) for 24 h and with Hyp (10 μg/ml) for 72 h. Total RNA was extracted and reverse transcribed into cDNA. Reactions were set up for real time PCR manufacturer's instructions (cDNA 2 µL, SYBR green mix 10 µL, 1 µL each forward or reverse primer (10 µM stock), 6 µL distilled water. The following primers were used: Bax, forward (5′-GGC GAT GAA CTG GAC AAC-3′) and reverse (5′-CCA AGG CAG CAG GAA GC-3′); Bal-2, forward (5′-GGC ATC TTC TCC TTC CAG-3′) and reverse (5′-CCC AGC CTC CGT TAT CC-3′); caspase-3, forward (5′-AGA TGT GGC TCT GTC C-3′) and reverse (5′-TGT GCT GTG GTC CTT-3′); caspase-9, forward (5′-GCA GTT GTG GGC GTT TC-3′) and reverse (5′-AGA GGC AGG AGG ATT GTT-3′); CytC, forward (5′-GGC TGC TGG ATT CTC-3′) and reverse (5′-GGC GAC ACC CTC ATA-3′); and β-actin, forward (5′-ACC CCG TGC TGC TGA CCG AG-3′) and reverse (5′-TCC CGG CCA GCC AGG TCC A-3′). Relative expression ratios were calculated according to the 2^−△△Ct^ method. Each sample was analyzed in quadruplicate.

### Experimental Animals and Group

Male Sprague Dawley (SD) rats (6–7 weeks old, 180–200 g) were purchased from SLAC Laboratory Animal Company (Shanghai, China) and housed in an animal facility with constant temperature (20–25°C), humidity (40–50%), and 12 h day and night switch light. Animals were supplied with sterile food and water. The research plan was approved by Ethics Committee of Army Medical University, and it adheres to international, national, and/or institutional guidelines for humane animal treatment and complies with relevant legislation.

Rats were randomly divided into two groups: normal control group (NG) and diabetes group (DG). All animals were supplied with normal rodent diet during the first week's acclimatization (considered as week 0). Subsequently, rats in NG were fed with the normal diet, while those in DG were fed with high-fat rodent pellet diet (HFD) for the duration of 4 weeks. Rats in DG were intraperitoneally injected with streptozotocin (STZ, 25 mg/kg) dissolved in 0.1 mM citric acid/sodium citric buffer (pH = 4.6) once a week for 8 weeks (Srinivasan et al., 2005; Wang et al., 2014). Before the injection, rats were fasted for 12 h. Rats in NG were injected with equivalent volume of 0.1 mM citric acid/sodium citric buffer solution. Next, DG rats were randomly divided into five groups: DG group; DG rats treated with 20 mg/kg Hyp (low-dose group, LG); DG rats treated with 50 mg/kg Hyp (medium-dose group, MG); DG rats treated with 100 mg/kg Hyp (high-dose group, HG), and DG rats treated with 50 mg/kg calcium dobesilate (positive control group, PG), respectively. Excluding the number of dead or unqualified blood glucose level animals in the process of modeling, the number of animals in each group was equal to 10 (n=10). Rats were daily treated with Hyp by intra-gastric administration once a day for additional 8 weeks. All animals were fed continuously according to their original feeding conditions during the whole experiment and sacrificed at the end of 20 weeks.

### Blood Glucose and Histological Analysis

Blood glucose levels were monitored monthly using a blood glucose meter (Roche, Diagnostics, Mannheim, Germany). After sacrificed, eyeballs were quickly removed. The anterior segment and lens were removed, the retinal tissue around the papilledema was separated. The samples were fixed, dehydrated, and paraffin-embedded. HE staining was carried out to generate slices for light microscopy. The pathological changes of the retinas were analyzed.

### Cell Viability and Cell Cycle Analysis

After treatment, retinas were isolated from all rats. RVECs was isolated from the retinas of rats in each groups as described above. Then, RVECs were seeded in 96-well plates pre-coated with fibronectin and cultured for 3 days. Cell viability was determined by MTT assay. In the cell cycle analysis, RVECs were harvested on day 3 and fixed in cold 70% ethanol for 18 h. After washing with PBS, cells were stained with propidium iodide (PI)/RNase dying solution (50 μg/ml PI and 10 μg/ml RNase). Cell cycles were analyzed on a BD FACSCalibur™ flow cytometer (BD Biosciences, CA, USA).

### Cell Apoptosis

Cell apoptosis was detected with the TUNEL assay using an *in situ* cell death detection kit (Roche Diagnostics, 12156792910). The images were taken with a fluorescence microscope (Olympus) and analyzed by Image J software. Apoptotic cells were determined by direct visualization by fluorescence microscopy in three independent experiments. The percentage of apoptotic cells is equal to the number of apoptotic cells/(the number of normal cells + the number of apoptotic cells).

### mRNA Expression by Real-Time RT-PCR in the *In Vivo* Experiment

RVECs derived from each group of rats were collected. Real time PCR reaction was made as described above. Briefly, total RNA was extracted and reverse transcribed into cDNA. Reactions were set up for real time PCR manufacturer's instructions. Relative expression ratios of Bax, Bcl-2, CytC, caspase-9, and caspase-3 were calculated according to the 2^−△△Ct^ method. Each sample was analyzed in quadruplicate.

### Protein Expression by Western Blot in the *In Vivo* Experiment

RVECs derived from each group of rats were collected and lysed in 100-μl RIPA buffer containing cOmplete™ Protease Inhibitor Cocktail (Roche, 11873580001). The cell lysates were centrifuged at the 10,000*g* for 20 min at 4°C, and the supernatant was kept for the Western blot assay. BCA method was used to determine the protein concentration in each cell lysate sample. 30 μg of protein was applied on SDS-PAGE. The protein gels were transferred onto PVDF membranes. The membranes were incubated with primary antibody anti-Bax (1:500), anti-Bcl-2 (1:500), anti-Cyto C (1:500), anti–caspase-3 (1:500), anti–caspase-9 (1:500) overnight at 4°C, and then incubated with secondary antibodies HRP-conjugated goat anti-rabbit IgG (1:2000) or HRP-conjugated goat anti-mouse IgG (1:5000) at room temperature for 1 h. Protein was detected using ECL system (Roche) and normalized by β-actin.

### Statistical Analysis

Statistical analysis was performed with SPSS18.0 software. Data were represented by mean ± SD, analyzed by one-way ANOVA for cell viability, cell cycle analysis, cell apoptosis, and Western blotting. Unpaired T-test was used for blood glucose levels.

## Results

### Isolated Cells Consist of a Nearly Pure Population of Endothelium Origin

Immuno-fluorescence staining of vWF, a specific marker expressed on vascular endothelium was performed on all our cultured RVECs (Boeri et al., 1994). An example of such staining is shown in **Supplementary Figure S1**. Homogeneous staining of vWF can be seen on the cultured RVECs, indicating these cells consist of a nearly pure population of endothelium origin.

### Hyp Reduces the Damage of RVECs Viability Induced by High Glucose *In Vitro*

**Figure 1:**
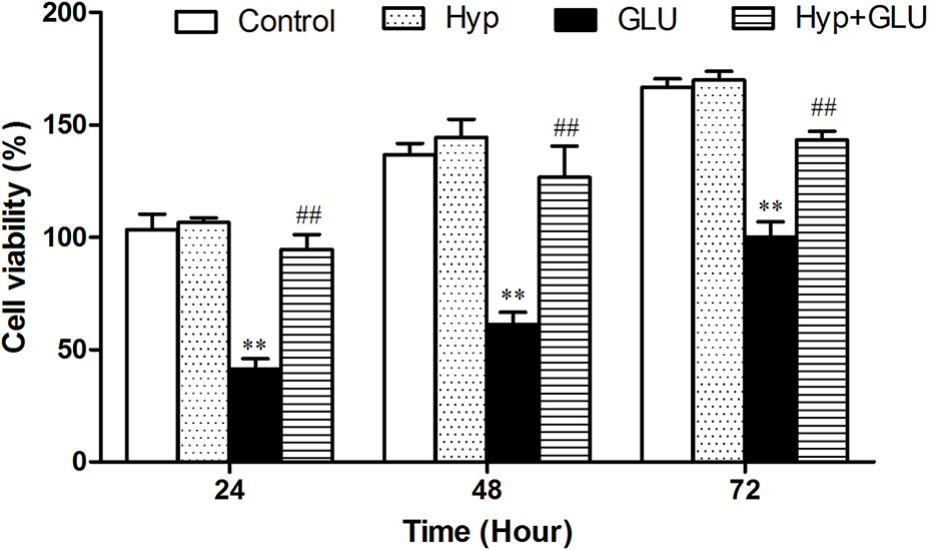
The effect of Hyp on RVECs viability treated by high glucose (n=6). The viability of RVECs was measured by MTT assay. RVECs were adherent cultured in 96-well plates. The cells were pre-treated with high glucose (30 mmol/L) for 24 h and incubated with Hyp (10 μg/ml) for 72 h. At 24, 48, and 72 h, respectively, 20 μl MTT solution was added and incubated for 4 h under dark. Thereafter, the supernatant was removed, DMSO (150 μl/well) was added to dissolve the produced formazan crystals. The absorbance was measured at 490 nm by multi-label Counter. To measure the cell viability as a percentage, the absorbance of each sample was divided into the absorbance of the control and multiplied by 100. **P < 0.01 compared to Control; ^##^P < 0.01 compared to GLU. GLU, high glucose; Hyp, hyperoside.

The effect of Hyp on RVECs viability treated by high glucose (GLU) using MTT assay. As shown in **Figure 1**, Hyp only had no influence on RVECs viability compare to control group. GLU incubation could decrease the viability of RVECs at the different time points after treatment. Hyp treatment significantly reduced the damage of cell viability induced by GLU.

### Hyp Increases the SOD Activity, Reduces MDA and ROS Level of RVECs Induced by High Glucose *In Vitro*

**Figure 2:**
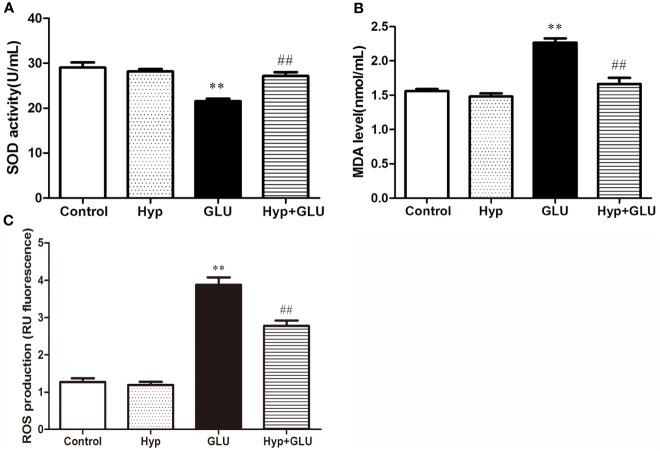
The effect of Hyp on SOD activity and MDA level of RVECs induced by high glucose (n=6). RVECs were adherent cultured in 96-well plates. The cells were pre-treated with high glucose (30 mmol/L) for 24 h and incubated with Hyp (10 μg/ml) for 72 h. The supernatant of cell culture were collected. The SOD activity **(A)** and MDA level **(B)** in the supernatant of RVECs was measured according to the kit operation instruction. The ROS production **(C)** was detected using DCFH-DA by flow cytometry. **P < 0.01 compared to Control; ^##^P < 0.01 compared to GLU.

SOD is an antioxidant metal enzyme and plays an important role in anti-oxidative system. MDA is a biomarker of oxidative stress. To investigate the effect of Hyp on oxidative damage of RVECs induced by GLU treatment, the SOD activity and MDA level in the supernatant of cell culture was measured. The results showed that GLU treatment significantly decreased SOD activity and increased MDA level in the supernatant of RVECs. The activity of SOD in the Hyp + GLU group is higher than that in the GLU group (**Figure 2A**). Hyp treatment significantly decreased the MDA (**Figure 2B**) and ROS (**Figure 2C**) level of in the supernatant of RVECs induced by GLU.

### Hyp Reduces the Changes of Apoptotic Proteins and Anti-Apoptotic mRNA Expression Induced by High Glucose *In Vitro*

**Figure 3:**
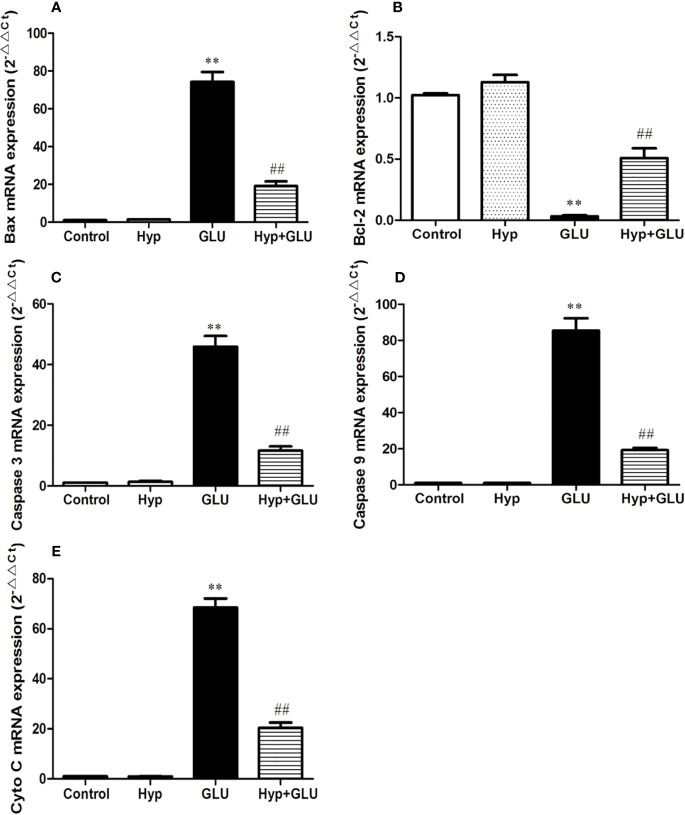
The effect of Hyp on the relative expression of caspase-3, caspase-9, Bax, Cyto-C, and Bcl-2 mRNA. Cultured cells in 6-well plates were pre-incubated with high glucose (30 mmol/L) for 24 h and with Hyp (10 μg/ml) for 72 h. Total RNA was extracted and reverse transcribed into cDNA. Reactions were set up for real time PCR manufacturer's instructions. Expression ratios were calculated according to the 2^−△△Ct^ method. Each sample was analyzed in quadruplicate. **(A)** Bax mRNA; **(B)** Bcl-2 mRNA; **(C)** caspase-3 mRNA; **(D)** caspase-9 mRNA; **(E)** Cyto C mRNA. **P < 0.01 compared to Control; ^##^P < 0.01 compared to GLU.

Caspase 3, caspase 9, Bax, and Cyto-C are several representative apoptosis regulatory proteins, Bcl-2 is a typical anti-apoptotic protein. And their expressions are closely correlated with cell apoptosis. By real-time PCR assay, the mRNA expression of pro-apoptotic and anti-apoptotic protein induced by GLU were measured. The results showed that GLU could increase the mRNA expression of these pro-apoptotic proteins and decrease the expression of anti-apoptotic protein. Treatment with Hyp significantly inhibited up-regulation of caspase 3, caspase 9, Bax, and Cyto-C mRNA and down-regulation of Bcl-2 mRNA induced by GLU (**Figure 3**).

### Hyp Treatment Reduced the Blood Glucose Level and Pathological Damage of Retina in Diabetic Retinopathy Model Rat

**Figure 4:**
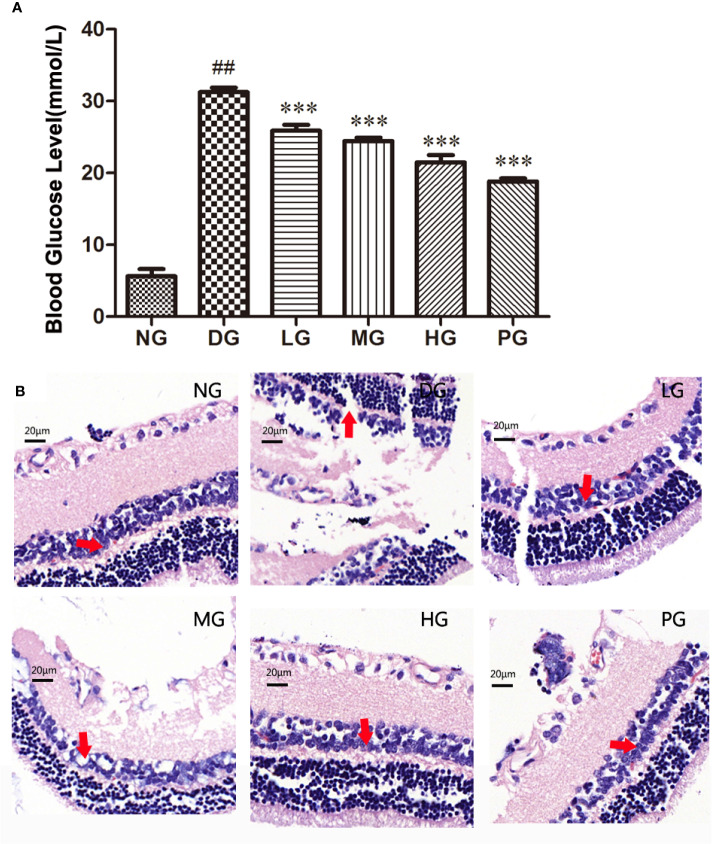
Effect of Hyp on the blood glucose level and histology analysis of the retina in diabetic retinopathy rats (n=10). **(A)** Effect of Hyp on the blood glucose level of diabetic rats. **(B)** Histology analysis of the retina in diabetic rats. NG: rats were fed with the normal diet; DG: rats were fed with high-fat rodent pellet diet for 4 weeks and intraperitoneally injecting with streptozotocin (STZ, 25 mg/kg) once a week for 8 weeks. LG: DG rats were treated with 20 mg/kg Hyp; MG: DG rats were treated with 50 mg/kg Hyp; HG: DG rats were treated with 100 mg/kg Hyp; PG: DG rats were treated with 50 mg/kg calcium dobesilate. Rats in LG, MG, HG or PG were treated with Hyp or calcium dobesilate once a day for additional 8 weeks. All animals were sacrificed at the end of 20 weeks. ^##^P < 0.01 compared to Control; ***P < 0.001 compared to DG.

To investigate the effect of Hyp on diabetes - induced damage of retinas, we established a diabetic retinopathy rat model by feeding the rats with HFD and injecting with STZ. The result showed that diabetic rats had high blood glucose level (over 11.1 mM) and displayed syndromes including excessive drinking, eating, and urination (data not shown). As shown in **Figure 4A**, the blood glucose level in diabetic retinopathy rat was much higher than that in normal control rats. Hyp treatment could decrease blood glucose levels in diabetic rats in a dose-dependent manner. Hyp has been found to increase glycolysis and decrease gluconeogenesis in STZ-induced hyperglycemia (Verma et al., 2013). In this study, diabetic rats were divided into groups and treated with Hyp at different doses (no treatment-DG, 20 mg/kg-LG, 50 mg/kg-MG, and 100 mg/kg-HG, respectively) for 2 months. As a positive control, a group of diabetic rats were treated with 50 mg/kg calcium dobesilate (PG). Histology analysis showed that the pathological changes of retina in the model rats were charactered by the obvious retinal edema, retinal detachment, subretinal basement membrane thickening. Compared with control group, the number of pericapillary cells decreased significantly, accompanied by interstitial hemorrhage, fibrosis, vitreous degeneration, and so on. After treatment with different doses of Hyp, the retinal damage was reduced, and the number of pericapillary cells was increased compared with the model group (**Figure 4B**). However, calcium dobesilate (50 mg/kg) appeared to elicit a larger reduction in the blood sugar level compared with 100 mg/kg Hyp.

### High-Dose Hyp Enhanced the Cell Viability and Promoted the Cell Proliferation in Diabetic Retinopathy Model Rat

**Figure 5:**
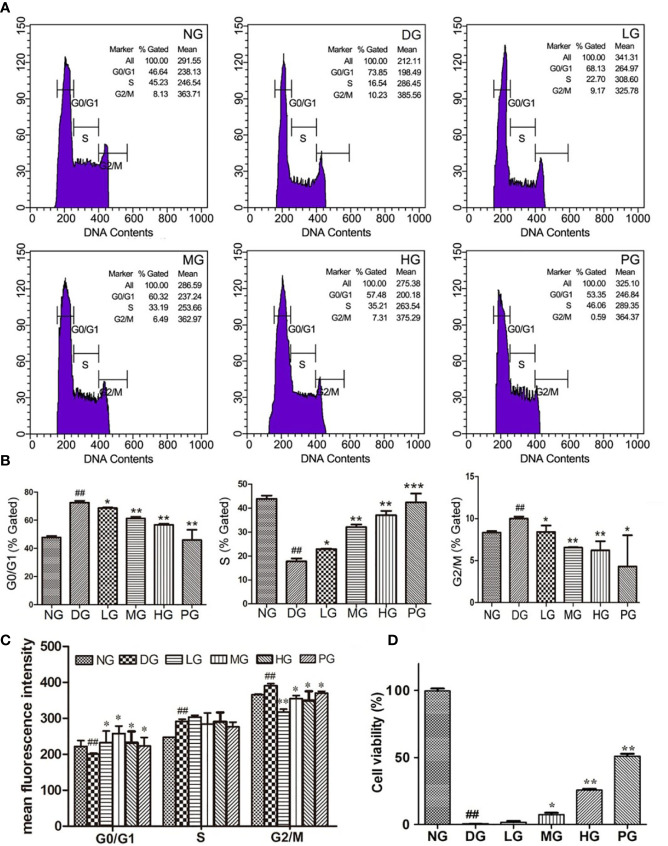
Effect of Hyp on cell viability and cell cycle analysis of the retinal vascular endothelium cells (n=6). RVECs derived from each group of rats were harvested and fixed in cold 70% ethanol for 18 h. After washing with PBS, cells were stained with PI/RNase dying solution. Cell cycles were analyzed on a BD FACSCalibur™ flow cytometer. **(A)** Photograph of effect of Hyp on cell cycle of cultured RVECs by flow cytometry. Statistical analysis of effect of Hyp on % gated **(B)** and mean fluorescence intensity **(C)** of cell cycle in cultured RVECs. **(D)** Effect of Hyp on cell viability of cultured RVECs by MTT assay. ^##^P < 0.01 compared to NG; *P < 0.05, **P < 0.01, ***P < 0.001 compared to DG.

To further determine the function of Hyp on vascular endothelium, primary RVECs were isolated from diabetic rats without treatment or with treatment of different doses of Hyp. The replication status on the cultured RVECs was evaluated by flow cytometry. As shown in **Figures 5A, B** the “% gated” of G_0_/G_1_ or G_2_/M phase in RVECs treated with Hyp prominently decreased compared with RVECs in DG. And Hyp treatment increased the percentage of S phase RVECs in DG rats. It was similar to calcium dobesilate-mediated effect on RVECs. Furthermore, Hyp treatment significantly increased the mean fluorescence intensity of G_0_/G_1_ phase cells and reduced the mean fluorescence intensity of G_2_/M phase cells, but had no influence on S phase cells (**Figure 5C**). Meanwhile, we determined the cell viability of RVECs isolated from diabetic rats. The cell viability was obvious increasing in RVECs from DG rats treated by Hyp compared with that in the cells from DG rats (**Figure 5D**).

### Hyp Alleviated Cell Apoptosis Level From Histological Aspect

**Figure 6:**
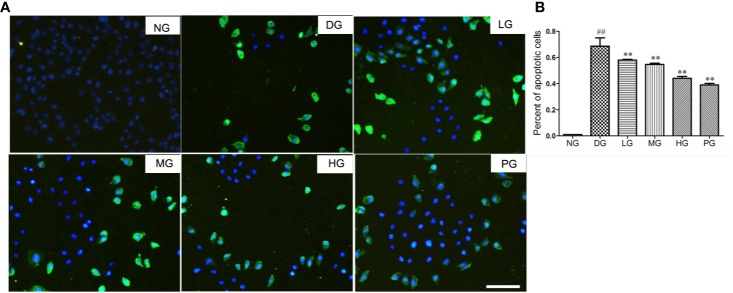
The effect of Hyp on cell apoptosis of RVECs from diabetic rats (n=6). Cell apoptosis was detected with the TUNEL assay using an *in situ* cell death detection kit. RVECs derived from each group of rats were harvested and fixed after 3 days' cultivation according to the manufacture instruction. The images were taken with a fluorescence microscope and analyzed by Image J software. **(A)** Photograph of effect of Hyp on cell apoptosis of cultured RVECs. **(B)** Percentage of apoptotic cells on cultured RVECs treated by Hyp. ^##^P < 0.01 compared to NG; **P < 0.01 compared to DG.

To reveal the mechanism of the protective effect of Hyp on diabetic retinopathy, TUNEL staining was used to detect the cell apoptosis of primary RVECs isolated from diabetic rats without treatment or with treatment of different doses of Hyp. Treatment with Hyp significantly inhibited cell apoptosis compared with DG in a dose-dependent manner (**Figures 6A, B**). Calcium dobesilate, as positive control, had similar result.

### Hyp Leads to the Down-Regulation of Apoptotic Proteins and Up-Regulation of Anti-Apoptotic Proteins Expression in Diabetic Retinopathy Model Rat

**Figure 7:**
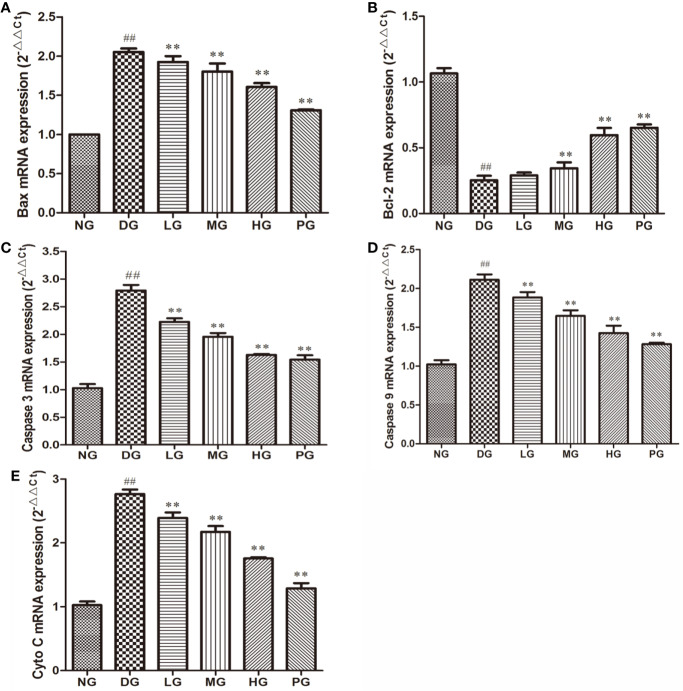
The effect of Hyp on apoptotic and anti-apoptotic mRNA expression of RVECs from diabetic rats (n=4). RVECs derived from each group of rats were harvested in the plates and cultured for 3 days. Total RNA was extracted and reverse transcribed into cDNA. Reactions were set up for real time PCR manufacturer's instructions. Relative expression ratios were calculated according to the 2^−△△Ct^ method. Each sample was analyzed in quadruplicate. **(A)** Bax mRNA; **(B)** Bcl-2 mRNA; **(C)** caspase-3 mRNA; **(D)** caspase-9 mRNA; **(E)** Cyto C mRNA. ^##^P < 0.01 compared to NG; **P < 0.01 compared to DG.

**Figure 8:**
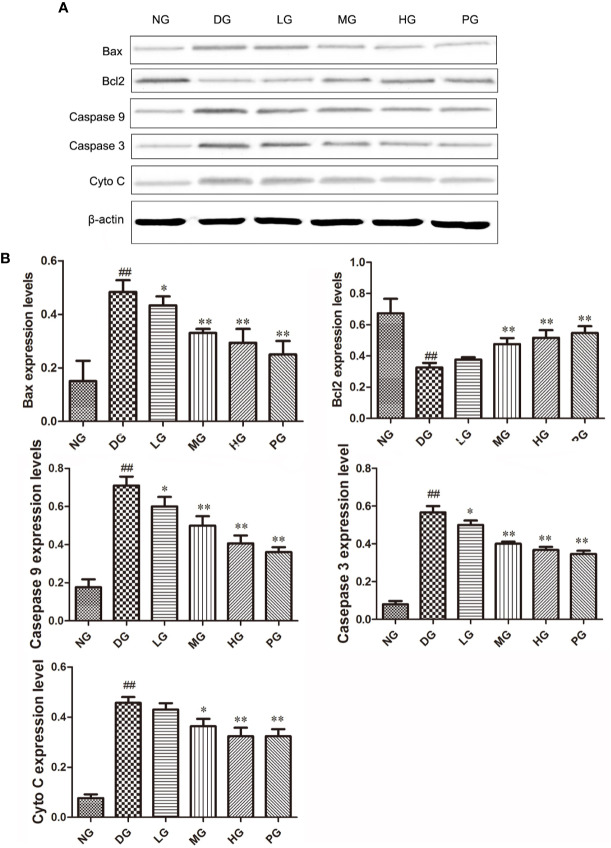
The effect of Hyp on Bax, Bcl-2, Cyto C, caspase-3, caspase-9 expression of RVECs from diabetic rats (n=4). **(A)** Photograph of protein expression by western blot. **(B)** Statistical analysis of protein expression by western blot. RVECs derived from each group of rats were collected and lysed in 100 μl RIPA buffer containing cOmplete™ Protease Inhibitor Cocktail. The cell lysates were centrifuged and the supernatant was kept. The protein concentration was determined by BCA method. 30 μg of protein was applied on SDS-PAGE. The protein gels were transferred onto PVDF membranes. Primary antibody anti-Bax, anti-Bcl-2, anti-Cyto C, anti–caspase-3, anti–caspase-9 (1: 500, overnight at 4 ℃), secondary antibodies HRP-conjugated goat anti-rabbit IgG (1:2000) or HRP-goat anti-mouse IgG (1:5000). Protein was detected using ECL system and normalized by β-actin. ^##^P < 0.01 compared to NG; *P < 0.05, **P < 0.01 compared to DG.

To confirm the effect of Hyp on apoptosis, we detected the levels of apoptotic proteins from the RVECs from diabetic rats without or with different doses Hyp treatment. The results of real time PCR (**Figure 7**) and Western blot (**Figure 8**) showed that Hyp could decrease the levels of caspase-3, caspase-9, Bax, and Cyto-C in DG model rat in a dose-dependent manner. In contrast, anti-apoptotic protein Bcl-2 level was increased at medium-dose and high-dose Hyp treatment groups. These changes were consisted with the calcium dobesilate-treated group (PG). These results suggest that Hyp promotes proliferation possibly through inhibition of apoptosis of the RVECs.

## Discussion

Hyp extracts from hypericum or cartages and has shown anti-oxidative, anti-inflammatory effects. However, its effect and mechanism on diabetic retinopathy has not been reported. In the present study, using a rat STZ–induced diabetic retinopathy model and rat retinal vascular endothelial cells, we initially investigated the therapeutic effect of Hyp on ameliorating the diabetes-induced damage of retinas. Our results showed that Hyp had the potential protective effects on diabetic retinopathy through anti-oxidation, inhibiting cell damage and apoptosis of RVECs induced by high glucose.

Diabetic retinopathy is recognized to be a microangiopathy occurring with diabetes, which is characterized by blood retinal barrier (BRB) breakdown, capillary basement membrane thickening, loss of pericytes, and the development of acellular and occluded capillaries (De La Cruz et al., 2004). Diabetes is known as a systematic metabolic disease associated with elevated inflammation (Brooks-Worrell and Palmer, 2012) and reactive oxygen species (ROS) generation (Igarashi et al., 1998; Kaneto et al., 2010). Our previous results also showed that Hyp had anti-oxidative effect on liver injury both *in vivo* and *in vitro* (Xing et al., 2011; Xing et al., 2015). Previous reports demonstrated that Hyp reduced the blood glucose level in a dose-dependent manner (Verma et al., 2013). This infusive effect inspired us to study its role on retinopathy. These results are consistent with our present findings about increasing the SOD activity, reducing ROS, MDA, and blood glucose level.

The diabetic retinopathy model by high-fat diet combined with STZ injection method is a classical model (Maines et al., 2006; Zhou and Zhou, 2007). The STZ-induced diabetic rat model displays morphologic and functional changes in the retinal vasculature similar to those observed in the early stage of human DR (Yu et al., 2001). To ensure the success of the model, fasting blood glucose (FBG) of all DG rats was measured regularly before Hyp administration. The average level of FBG of all DG rats, which were included in the final randomized grouping and administration, was higher than 20 mM. And those rat whose FBG was lower than this standard were excluded. Our results suggested that Hyp could decrease the FBG level of DG rat, and reduce its retinal damage and increase the number of pericapillary cells compared with the model group. Therefore, treatment of Hyp in diabetic rats has been demonstrated the potential improvement of retinopathy.

Calcium dobesilate has been used to treat diabetic retinopathy at the systematic and local ocular level with promising results. In present study, it was used as positive control drug to evaluate the experiment system and protective extent of Hyp on diabetic retinopathy. However, it is slightly disappointing that Hyp (100 mg/kg) did not show better efficacy than calcium dobesilate (50 mg/kg) in decreasing blood glucose level and reducing retinopathy.

Apoptosis is an major contributor to neuronal cell death in the early course of diabetic retinopathy (Zhang et al., 2008). In early diabetic retinopathy, RVECs apoptosis mainly contributes to BRB breakdown (Xu et al., 2018). Our previous study found that Hyp could antagonize rat retinal vascular endothelial cell apoptosis induced by oxidative damage (Wu et al., 2013). In the present study, we have demonstrated that there is no significant difference in cell viability between Hyp treated group and control group at the same time-point by the MTT assay. Therefore, we think that the effect of Hyp on cell proliferation is not directly stimulation, but Hyp can reduce the damage of RVECs viability induced by high glucose. This is a part of the protective mechanism of hyperoside on diabetic retinopathy. Furthermore, we have also demonstrated that Hyp treatment significantly inhibited RVECs apoptosis of DG rat in a dose-dependent manner. The results of qPCR and Western blot showed that Hyp treatment leaded to the down-regulation of apoptotic proteins (caspase-3, caspase-9, Bax, and Cyto-C) and up-regulation of anti-apoptotic proteins (Bcl-2) expression. During cell apoptosis, caspase-8 and -9 play the role of the initiator to in response to proapoptotic signals. Caspase-3 is as the effector, which through the cleavage of several vital proteins finally induce an apoptotic phenotype. Cleaved-caspase-3 is the active form of caspase-3. As several representative molecules of apoptosis signaling pathway, we detect the expression of caspase-3 and caspase-8 as representatives in this paper, but not to observe cleaved-caspase-3 and cleaved caspase-8. However, we also detected some other apoptotic signal molecules, such as Bax, Bcl-2, and CytoC, to prove the effect of hyperoside on apoptotic signal. The rate of cell viability and S phase cells in the RVECs from DG rats treated with Hyp was also higher than that in the RVECs of DG group in the dose dependent manner. These results suggest that the protective effect of Hyp on the diabetic retinopathy was closely related with inhibiting cell damage and apoptosis of RVECs induced by high glucose.

Hyp has shown to prevent glomerular podocyte apoptosis in STZ-induced diabetic nephropathy (Zhou et al., 2012). In addition, Hyp was reported to attenuate inflammation in human umbilical vein endothelial cells (Sun et al., 2002). Endothelial cells subjected to the oxidative damage can be protected by Hyp treatment through its anti-apoptosis pathways such as regulating SIRT-1 and Bcl-2 family (Zhang et al., 2008), regulating Mcl-1 and Bid (Liu et al., 2016), or mediation of P38 expression (Hao et al., 2016). These findings are fairly similar with our findings. Therefore, further study is needed to illustrate the beneficial effect of Hyp in treatment of human diabetes. It has been reported that Hyp can inhibit apoptosis induced by hydrogen peroxide through p38/c-fos, thus protecting umbilical vein endothelial cells (Hao et al., 2016). Our results also showed that Hyp attenuated hydrogen peroxide-induced L02 cell damage *via* MAPK-dependent Keap1–Nrf2–ARE signaling pathway (Xing et al., 2011) or GSK-3β Inactivation (Xing et al., 2015). As known to all, these pathways are also closely related to cell apoptosis and proliferation. Therefore, we speculate that feedback regulation between MAPK-Akt and GSK3β signaling pathways may be a potential target for Hyp in the treatment of diabetic retinopathy.

## Conclusion

In summary, our study investigates the therapeutic effects of Hyp on diabetic retinopathy. Administering Hyp in diabetic rats ameliorates the injury of retinas. The RVECs from diabetic rats show increasing of proliferation in histological and cell cycle analyses. The effects of Hyp in improvement of the RVECs could be due to its inhibitory effect on anti-oxidation, inhibiting cell damage and apoptosis induced by high glucose. Our data indicate that Hyp may be a potential candidate for drug development in the therapy of diabetic retinopathy.

## Data Availability Statement

All datasets presented in this study are included in the article/**Supplementary Material**.

## Ethics Statement

The animal study was reviewed and approved by Ethics Committee of Army Medical University.

## author contributions

WW and JC conceived, designed the present study. BL administrated and supervised the present study. WW and ZX developed the methodology, reviewed the manuscript. BL analyzed, and interpreted the data. QZ, YM, XB, and XY acquired the data. All authors reviewed the final manuscript. All authors declare that they have no competing interests.

## Funding

This work was supported by the National Natural Science Foundation of China (NO. 81303314). We also gratefully acknowledge the financial support from Science and Technology Achievements Transformation Fund Project of Third Military Medical University (No. 2015XZH19), National Science and Technology Major Project (NO. 2018ZX09J18109-005).

## Conflict of Interest

The authors declare that the research was conducted in the absence of any commercial or financial relationships that could be construed as a potential conflict of interest.

## Supplementary Material

Supplementary Figure S1 The expression of vWF on cultured RVECs. The characteristics of the cultured cells were determined on day 3 by immunofluorescence staining using anti-vWF antibody.
