# Supplementary figures and images for: Hyperoside Ameliorates Diabetic Retinopathy via Anti-Oxidation, Inhibiting Cell Damage and Apoptosis Induced by High Glucose
Source: Front Pharmacol. 2020 May 29;11:797. doi: 10.3389/fphar.2020.00797 (PMC7273924; doi:10.3389/fphar.2020.00797)

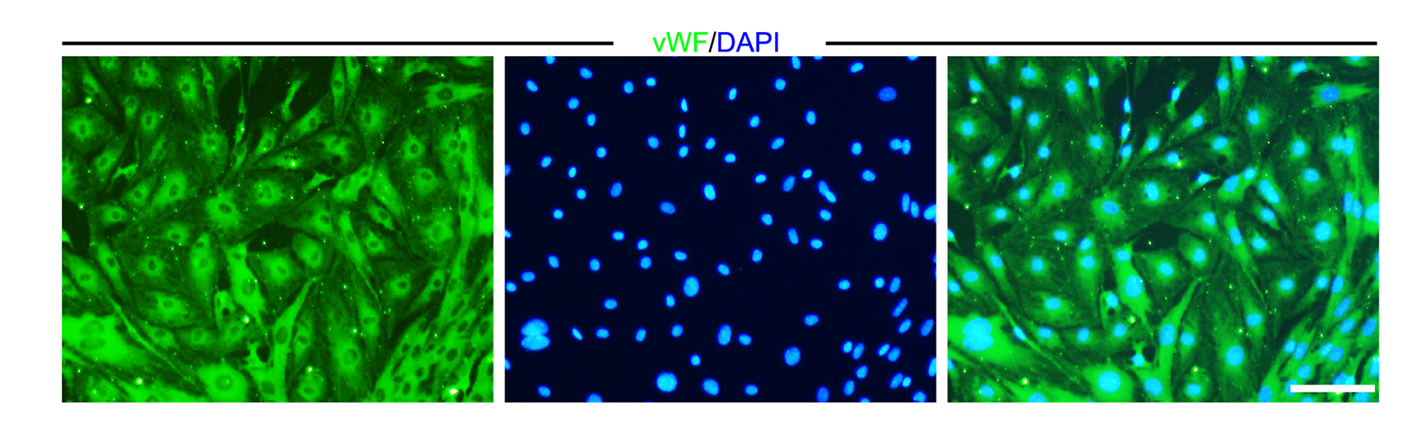

Supplement: Supplementary Figure S1 — The expression of vWF on cultured RVECs. The characteristics of the cultured cells were determined on day 3 by immunofluorescence staining using anti-vWF antibody. [file Image_1.tif]
